# Response to: Near-death experiences and the importance of transparency in subjectivity, ontology and epistemology

**DOI:** 10.1093/braincomms/fcab305

**Published:** 2022-02-12

**Authors:** Daniel Kondziella, Charlotte Martial

**Affiliations:** Department of Neurology, Rigshospitalet, Copenhagen University Hospital, Copenhagen 2100, Denmark; Department of Clinical Medicine, University of Copenhagen, Copenhagen 2100, Denmark; Coma Science Group, GIGA-Consciousness, University of Liège, Liège 4000, Belgium; Centre du Cerveau^2^, University Hospital of Liège, Liège, Belgium

We thank Dr Stripp for his interest in our paper^1^ and for sharing his thoughts about it in a sophisticated and polite manner. We have received a great number of critical comments about this paper, most of which were sent privately to the authors and most of which were much less civilized, testifying to the fact that near-death experiences (NDEs) trigger a profound interest and that associating them with a biological and evolutionary purpose appears to evoke strong emotions in many people.

In response to Dr Stripp, we like to reiterate a few of the following thoughts laid out in our paper.

There are no data to indicate that the phenomenology of NDEs differs in situations that are (i) associated with a threat to life and impaired brain physiology such as a cardiac arrest; (ii) associated with a threat to life but unimpaired brain physiology such as a near-miss traffic accident and (iii) associated with non-life-threatening situations such as drug abuse or fainting. Indeed, as pointed out by K.R. Nelson in his Scientific Commentary^2^ on our paper, ‘the term “near-death” borders on misnomer since in half the instances of near-death, individuals are not in medical danger’.^3^

The data that do exist indicate that NDEs in all three circumstances referred above are phenomenologically similar.^4–6^ From the phenomenology of the experience, one cannot tell whether what happened was a cardiac arrest or abuse of ketamine. This similarity suggests that also the brain mechanisms behind these experiences are similar, if not identical.

This would make sense because it is a prerequisite for someone being able to report an NDE that during the actual experience they have had sufficiently preserved cerebral function and have survived without major brain damage. Without a functioning brain, how would it be possible to make an experience so rich in details, store it over many years, retrieve it easily and report on it in an eloquent manner many years later?

Rather than concluding that NDEs made during cardiac arrest are evidence for human consciousness being able to exist outside the brain, the most parsimonious conclusion would be that NDEs are made just prior to the loss of consciousness—and hence can be remembered with successful resuscitation.

We certainly agree with Dr Stripp (and we say so in our paper) that our study is not absolute proof of the hypothesis that thanatosis is the evolutionary origin of NDEs. We also acknowledge the fact that we may never know for certain whether NDEs have solely a biological meaning or indeed may hint to the existence of an after-life. However, Dr Stripp’s critique about our use of anecdotal evidence, being no different from the anecdotal evidence used by proponents for a transcendental meaning of NDEs, does not seem justified. We use anecdotal evidence for thanatosis and NDE occurring in humans under attack by lions and other large predators to support our argument that such experiences indeed can occur in these circumstances: this can hardly be denied, unless one presumes that these people were lying about their experiences. In contrast, proponents of a transcendental meaning of NDEs use anecdotes to support their hypothesis that NDEs are evidence for humans being able to make conscious experiences without a functioning brain. This argumentation is more far-fetched because, as pointed out earlier, the assumption that NDEs are being made just before consciousness is lost is more parsimonious.

It appears to us that many people with a special interest in NDEs seem not to recognize that these are conscious experiences based on cerebral phenomena (albeit interesting ones), just like other subjective neurological experiences such as migraine aura or time–space synaesthesia. This lack of neurological understanding appears unfortunate but may reflect our observation that neuroscientific expertise is often underrepresented in people with an interest in NDEs, including researchers (many of the most prominent being cardiologists^7^ or anaesthesiologists).^8^

On a lesser note, we agree with Dr Stripp that NDEs differ from what most people would think of as hallucinations, but NDEs do fulfil the criteria for hallucinations as laid out by the *Diagnostic and Statistical Manual of Mental Disorders, 5th Edition (DSM-5)* of the American Psychiatric Association, i.e. hallucinations are ‘a sensory perception that has the compelling sense of reality of a true perception but that occurs without external stimulation of the relevant sensory organ’. In the same vein, how to define the term ‘predator’ can be discussed but this semantics is of little relevance to the point we are making.

Far more interesting to us is Dr Stripp’s suggestion that reflecting critically on one’s own subjectivity and stance is important for every researcher. It goes without saying that we fully support this notion, but we are curious how can it be that of all the papers published in *Brain Communications* it is exactly our NDE paper that triggers a comment about this very basic principle?

We have not investigated this matter, but we wonder whether this is because the spiritual values of many people interested in NDEs are at odds with an acceptance of the principles of evolution. Indeed, the conflict between proponents of an evolutionary model and those of a spiritual model is as old as Darwin’s theory itself.^9^ Again, we would like to refer to the Scientific Commentary of K.R. Nelson who gives Solomonic advice on how to reconcile these opposing views: ‘Here James offers counsel to persons whose near-death experience steadfastly transformed personal meaning and spirituality: “by their fruit ye shall know them, not by their roots”’.^2^

In conclusion, it appears to us that the field of NDE research is subject to a widely held belief that there is something fundamentally special, if not supra-natural, about NDEs, like the notion that conscious experiences can be made in the absence of a functioning brain. Although we cannot know what the future brings, we think that our hypothesis of thanatosis being the evolutionary origin of NDEs has a good chance to stand the test of time, just like so many other evolutionary hypotheses of behaviours and traits that have prevailed since Charles Darwin’s theory.^10^

## Data availability

Data sharing is not applicable to this article as no new data were created or analysed.

## Competing interests

The author reports no competing interests.
